# Non-invasive Technology Advances in Cancer—A Review of the Advances in the Liquid Biopsy for Endometrial and Ovarian Cancers

**DOI:** 10.3389/fdgth.2020.573010

**Published:** 2020-12-11

**Authors:** Mark R. Openshaw, Terri P. McVeigh

**Affiliations:** Cancer Genetics Unit, Royal Marsden NHS Foundation Trust, London, United Kingdom

**Keywords:** biomarker, circulating tumor (ctDNA), microRNA, DNA, circulating tumor cell (CTC)

## Abstract

Improving cancer survival rates globally requires improvements in disease detection and monitoring, with the aim of improving early diagnosis and prediction of disease relapse. Traditional means of detecting and monitoring cancers rely largely on imaging and, where possible, blood-based protein biomarkers, many of which are non-specific. Treatments are being improved by identification of inherited and acquired genomic aberrations in tumors, some of which can be targeted by newly developed therapeutic interventions. Treatment of gynecological malignancy is progressively moving toward personalized therapy, as exemplified by application of PARP-inhibition for patients with BRCA-deficient tubo-ovarian cancers, or checkpoint inhibition in patients with mismatch repair-deficient disease. However, the more recent discovery of a group of biomarkers described under the umbrella term of “liquid biopsy” promises significant improvement in our ability to detect and monitor cancers. The term “liquid biopsy” is used to describe an array of tumor-derived material found in blood plasma and other bodily fluids such as ascites, pleural fluid, saliva, and urine. It includes circulating tumors cells (CTCs), circulating nucleic acids including DNA, messenger RNA and micro RNAs, and extracellular vesicles (EVs). In this review, we discuss recent advancements in liquid biopsy for biomarker detection to help in diagnosis, prognosis, and planning of treatment of ovarian and endometrial cancer.

## Introduction

### Genomic Changes in Cancer Cells

Cancer is a ubiquitous disease, with 18 million new cases and 9.5 million cancer-related deaths reported annually worldwide (1). Cancer can be characterized by distinctive hallmarks, including abnormal growth control, escape of normal apoptotic pathways, growth factor secretion, and cell-cell interactions (2, 3). Cancer growth, invasion, and metastasis are facilitated by changes in two main classes of genes—tumor suppressor genes and proto-oncogenes. Tumor suppressor genes are a class of genes that work to inhibit cell proliferation, and loss of function of tumor suppressor genes is oncogenic. Proto-oncogenes are normal cellular genes that encode for protein products, working in concert with the products encoded by tumor suppressor genes to regulate cell growth and proliferation. Oncogenes, by comparison, are abnormal genes, the products encoded by which promote carcinogenesis, through over-activation, or over-production of signals promoting cell growth/proliferation. Proto-oncogenes may be converted to oncogenes by a variety of mechanisms, including acquisition of gain-of-function mutations, localized copy number variation (amplification), or chromosomal translocation; which lead to constitutive over-activation or over-expression of the protein product (2). Many oncogenes, closely related to normal cellular proto-oncogenes, have been identified in common viruses, and infection with such viruses may in turn promote tumourigenesis. So-called “oncogenic viruses” include Hepatitis B and Hepatitis C (both implicated in development of hepatocellular cancer), Epstein Barr virus (Nasopharyngeal carcinoma, Hodgkin, and Burkitt's lymphoma), Merkel cell Polyomavirus (Merkel cell cancer), HtlV-1 (adult T-cell Lymphoma), Human Herpes virus 8 (Kaposi's sarcoma), and Human Papillomavirus (cervical cancer, ano-genital cancer, some head and neck cancers) (4).

Cells acquire genomic aberrations during each mitotic division. Uncorrected, these aberrations will then be passed on to subsequent generations. Driver mutations are those which confer a growth and/or survival advantage to the cell. The subclone bearing a driver mutation will have enhanced fitness compared to other cells and will therefore be preferentially selected for proliferation. Acquisition of 2–8 driver mutations will result in development of cancer. The molecular evolution of cancer may be mapped in parallel to morphological changes, through different phases of dysplasia, early neoplasia and late neoplasia (5). The majority of genomic changes in cancer are acquired as somatic events, but a small proportion of affected individuals carry germline variants that may be inadvertently identified by testing of tumor-derived DNA (6). Molecular aberrations in cancer provide unique targets for treatment, such biomarkers thus representing companion diagnostics (7).

Despite increasing advances in technology and improvement in our understanding of tumor biology, a number of challenges exist. These include the difficulty of obtaining tumor biopsies from which DNA of appropriate quality and quantity can be used for genomic analysis, the presence of intra-, and inter-tumoral genomic heterogeneity (8) which hinders both our understanding of tumors (9) and response to targeted agents (10), and dynamic change in tumors over time, particularly after treatment resulting development of resistance (11). Therefore, developments of circulating biomarkers that can measure this spatial and longitudinal heterogeneity are highly sought after.

The three main gynecological cancers include cervical, ovarian, and endometrial cancer. Major advances have been made in prevention of and screening for cervical cancer. Cytology-based population-level screening has existed for many decades in many developed countries, supplemented, or supplanted in more recent years with Human Papillomavirus (HPV) testing (12, 13). Vaccination against oncogenic HPV infection has resulted in significant reduction in the number of high grade cervical lesions identified in young women (14), and the World Health Organization has endorsed vaccination as one of the key strategies to try to eradicate cervical cancer as a public health problem, with a view to achieving 90% vaccine uptake in girls before age 15 (15, 16). Compared to cervical cancer, no such preventative vaccine programme exists, as yet, for endometrial or ovarian cancer, although much research has focused on development of vaccine for Lynch syndrome-related malignancies (17–19).

Endometrial cancer is the most common cancer of the female reproductive tract, and its incidence is increasing, a fact largely attributed to increasing rates of obesity, and trends in exogenous estrogen use (20, 21). Endometrial cancer most often presents early, with irregular bleeding per vaginum. Comparatively, long heretofore known as the “silent killer,” most cases of ovarian cancer are diagnosed at late stages, as the presenting symptoms are often non-specific (22). At present, no screening test has been conclusively proven to impact mortality of ovarian/endometrial cancer in women at population-level or higher risk.

### Heritable Cancer Predisposition Syndromes

A number of heritable cancer predisposition syndromes are associated with significantly increased lifetime risks of ovarian and/or endometrial cancer (Table 1).

**Table 1:** High risk cancer predisposition syndromes associated with endometrial and/or ovarian cancer, associated gene, and other cancers.

**Associated gene**	**Ovarian cancer risk associated with germline pathogenic variant**	**Endometrial cancer risk associated with germline pathogenic variant**	**Other associated cancer types**
*BRCA1* (23, 24)	43–76%^a^	–	Breast, Male breast, Prostate, Pancreatic
*BRCA2* (23, 24)	7.5–34%^a^	–	Breast, Male breast, Prostate, Pancreatic, Melanoma
*RAD51C* (25)	6–21%^a^	–	Breast
*RAD51D* (25)	7–23%^a^	–	Breast
*BRIP1 (26)*	3.6–9.1%^a^	–	–
*PALB2 (27)*	2.4–9.7%^a^	–	Breast, Male breast, Pancreatic
*STK11 (28)*	~21%^b^	~9%	Colorectal, Breast, Stomach, Small bowel, Cervix (adenoma malignum), Pancreas, Testicular, Lung
*MLH1 (29)*	4.8–15.4%^c^	33.1–52.3%^c^	Colorectal, Stomach, Upper gastrointestinal, Hepato–pancreatico-biliary, Brain
*MSH2 (29)*	5.7–28%^c^	41.8–71.6%^c^	Colorectal, Stomach, Upper gastrointestinal, Hepato-pancreatico-biliary, Brain
*MSH6(29)*	0–31.2%^c^	27.3–65%^c^	Colorectal, Stomach, Upper gastrointestinal, Hepato–pancreatico-biliary, Brain
*PMS2 (30)*	–	7–24%^c^	Colorectal
*PTEN (31)*	–	17–39%^d^	Breast, Thyroid, Colorectal, Kidney, Skin
*POLD1 (32)*	–	Uncertain, moderate-high	Colorectal, Gastric,? others

a *High Grade Serous Ovarian cancer (33)*.

b *Sex-cord stromal tumors with annular tubules (34, 35), rarely oxyphilic sertoli cell cancers (36)*.

c *Usually non-serous ovarian cancer (37); Usually endometrioid endometrial cancer (38)*.

d *Endometrioid common, multiple different types reported (38)*.

### Risk-Reducing Strategies for Ovarian and/or Endometrial Cancer

At present, in the absence of a proven screening test, for women with such conditions, the only proven way of minimizing their risk of endometrial and/or ovarian cancer is to undergo prophylactic surgery. In most cancer predisposition syndromes associated with increased gynecological cancer risk, the average age at diagnosis far predates that in the general population, often prior to the average age of menopause. To mitigate this risk, the age at which prophylactic surgery is recommended (35–40 years) also predates the age at which natural menopause would ordinarily occur, by many years (39–41). If premenopausal oophorectomy is undertaken, sequelae include infertility and premature menopause, with the associated risks of early cardiovascular, cognitive, or bony adverse events (42).

As it is postulated that the majority of serous epithelial ovarian cancers begin in the fallopian tube (43), early salpingectomy with delayed oophorectomy has been proposed as an alternative to standard risk-reducing bilateral salpingo-oophorectomy in certain patients with increased risks of ovarian cancer (44). It is uncertain whether such an approach has a role in women with Lynch Syndrome, given that the proportion of ovarian cancer of tubal origin in this setting is less well-established.

For women with Lynch syndrome who are also at increased risk of colorectal malignancy, requiring biannual colonoscopy, colonic surveillance following hysterectomy may be more uncomfortable, and technically more difficult (45). For women with Lynch Syndrome who wish to defer surgical prophylaxis, surveillance with transvaginal ultrasonography, endometrial pipelle biopsy, and/or hysteroscopy is not recommended, as there is no conclusive evidence to suggest this approach leads to stage-shift or improved survival in high-risk individuals (41). For other endometrial cancer predisposition syndromes (e.g., Cowden/Peutz-Jeghers syndromes), there is a paucity of evidence about the role of surveillance vs. risk-reducing surgery, and risk-management may be complicated further by a high incidence of benign endometrial pathology in such conditions. For women at population-level endometrial cancer risk, the use of transvaginal sonography with endometrial biopsy as required as a screening tool is limited by a number of factors—including the intimate and invasive nature of the test, high cost, need for specialist sonographers, and the need for different cut-offs based on age and menopausal status (46). These issues have prompted discovery efforts for a non-invasive blood-based biomarker.

With respect to ovarian cancer surveillance, a multimodal strategy including transvaginal ultrasound, CA125, and considering factors such as age, menopausal status, and background predisposition has been shown to result in stage-shift at diagnosis of ovarian cancer in women at higher risk (47). However, whether such stage-shift translates to survival benefit has not yet been proven. CA125 has been included as a component of the ovarian cancer risk of malignancy index (RMI), alongside ultrasound scan result and menopausal status. An RMI score above 200 has been shown to be highly discriminate at detecting ovarian cancer (sensitivity 86%, specificity 97%) (48) in patients presenting with suspected symptoms. Major limitations of CA125 as a screening tool include observations that CA125 is raised in only 50–60% of patients with stage I and II cancers, while false positives in patients with no pathology/benign gynecological pathology are not infrequent (49). Whilst CA125 is raised in a higher proportion of advanced cases of ovarian cancer, and the measurement of CA125 combined with physical examination can detect up to 90% of relapses (50), there is evidence that commencing treatment based on elevated CA125 levels alone is not beneficial (51). Other putative blood-borne tumor biomarkers such as HE4, CHI3L1, PEBP4, and AGR2 have also been proposed, but have similar limitations when used individually, and even when multiplexed with other markers (52). The utility of such markers as part of multimodal strategies require further evaluation in large scale studies (53).

### Somatic Aberrations in Ovarian and Endometrial Cancers

**Figure 1:**
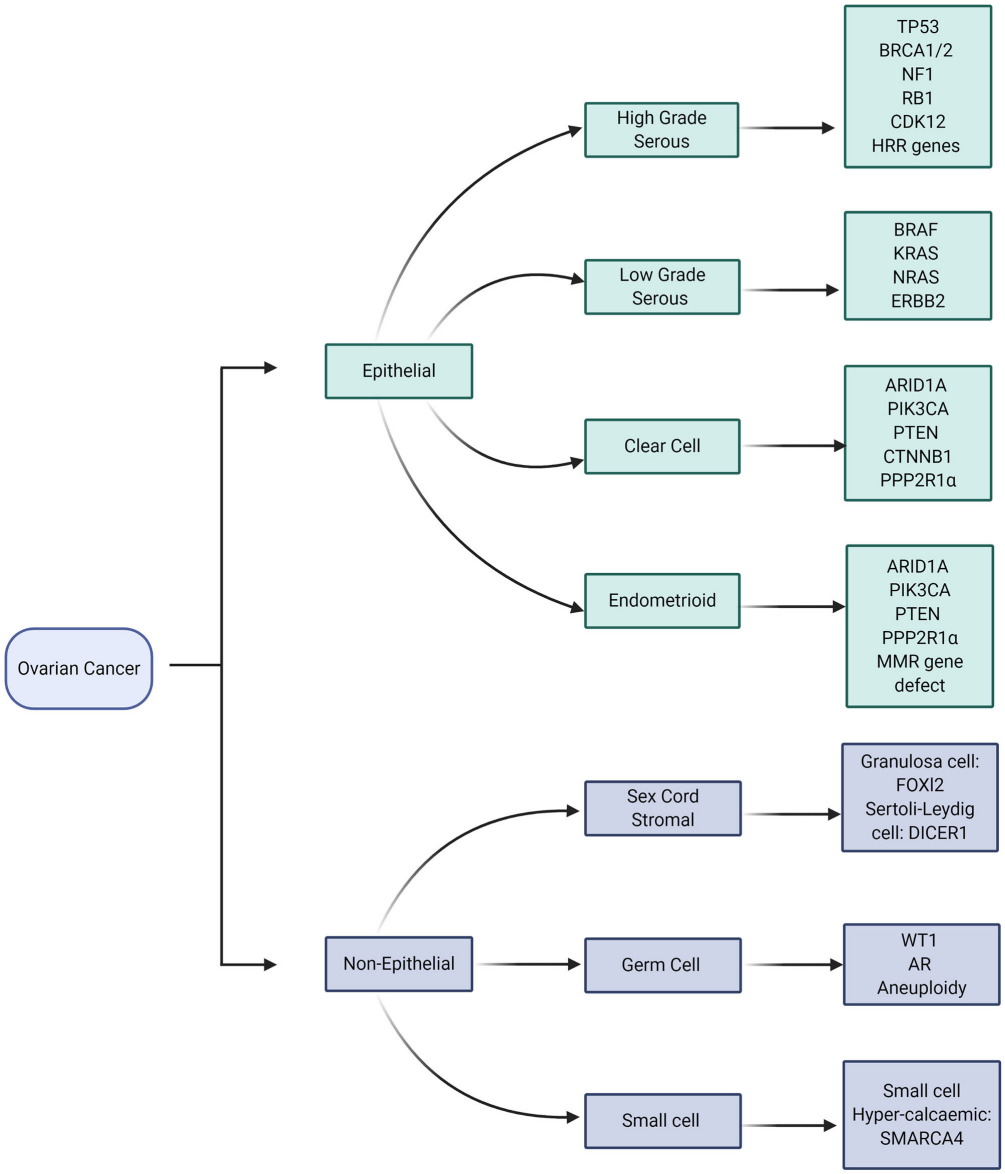
Molecular aberrations in different subtypes of Ovarian Cancer. Image created and exported form biorender.com under a paid subscription.

**Figure 2:**
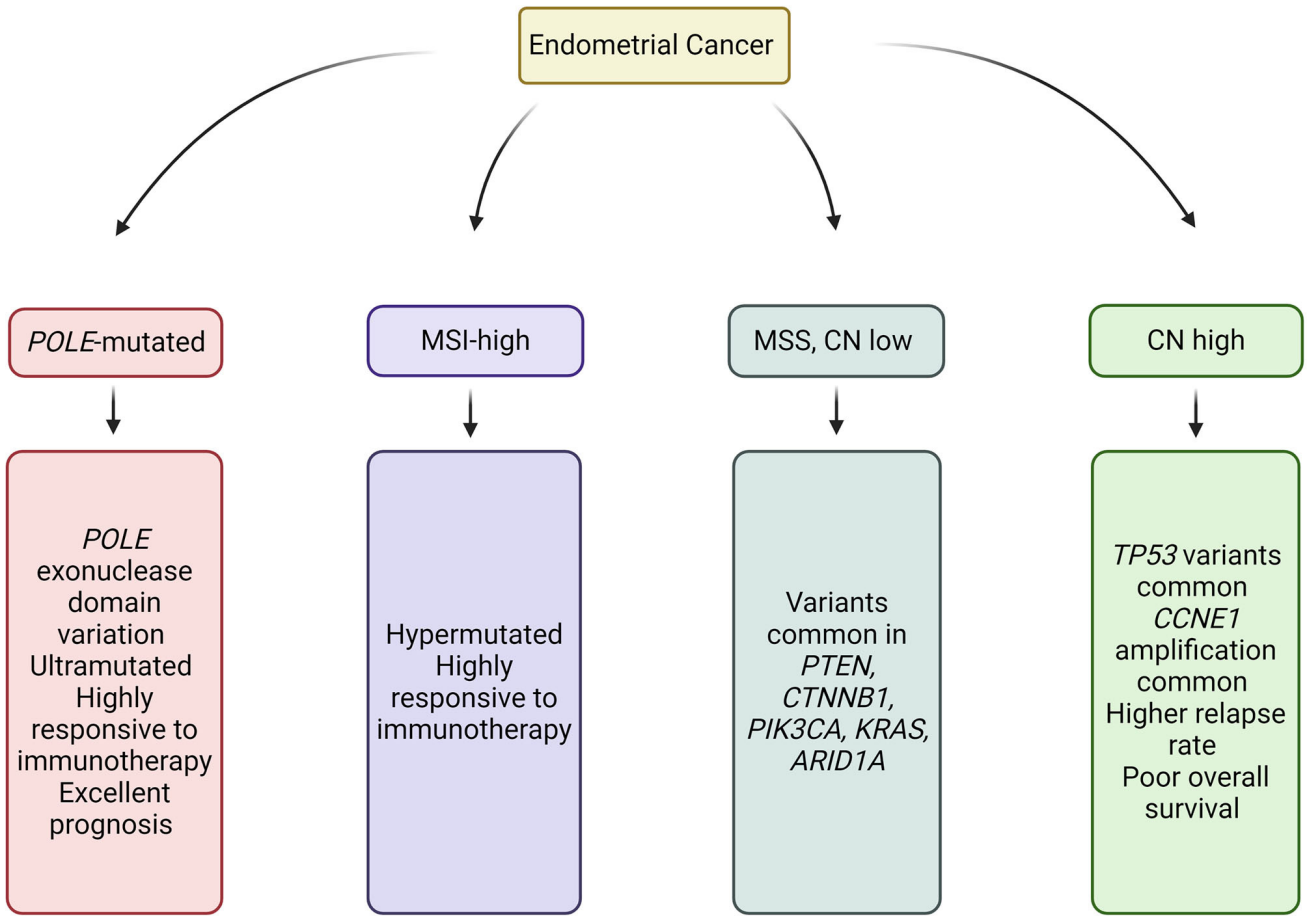
Molecular subtypes of Endometrial Cancer. Image created and exported form biorender.com. CN, Copy number; MSS, Microsatellite stable; and MSI, Microsatellite instability.

The molecular characteristics of ovarian cancers are very closely aligned to the histopathological subtype (Figure 1). *TP53* gene mutations are almost ubiquitous in High Grade Serous Ovarian Cancer (HGSOC) whilst variants in a number of other genes *NF1, BRCA1, BRCA2, RB1*, and *CDK12* are also commonly identified (Figure 2) (54). Approximately 41–50% of HGSOC demonstrates Homologous Recombination Repair deficiency by virtue of somatic or germline genomic defects, which may then be exploited by therapy, with platinum-based chemotherapy and PARP inhibition (55). There are a number of ways to define HR deficiency (56), all of which currently involve a tumor biopsy; therefore non-invasive routes to defining HR deficiency would be beneficial.

Characterization of endometrial cancer by integrated genomic, transcriptomic, and proteomic characterization has identified four sub-categories, which overlap but do not directly correlate with histopathological subtype (57) (Figure 2). Classification of cancer by molecular subtype rather than histological subtype is advantageous, in that it can direct therapy, provide prognostic information, and identify those patients requiring germline genetic assessment.

Mismatch Repair (MMR) -deficient (MMR-d) tumors, defined by absence of one or more MMR proteins by IHC analysis, usually demonstrate high levels of microsatellite instability (MSI-H), exhibit high expression of pro-inflammatory genes and enhanced neo-antigen expression (58). Approximately 10% of epithelial ovarian cancers (mostly non-serous) (59, 60) and 20–40% of endometrial cancers (57, 61) demonstrate features of mismatch repair deficiency. Several studies have shown favorable response rates and improved prognosis for patients with MMR-d tumors treated with immune checkpoint inhibitors (58, 62). A number of trials investigating the utility of checkpoint blockade in patients with Epithelial Ovarian and Endometrial cancer are underway (63–65).

## Aim of Review

**Figure 3:**
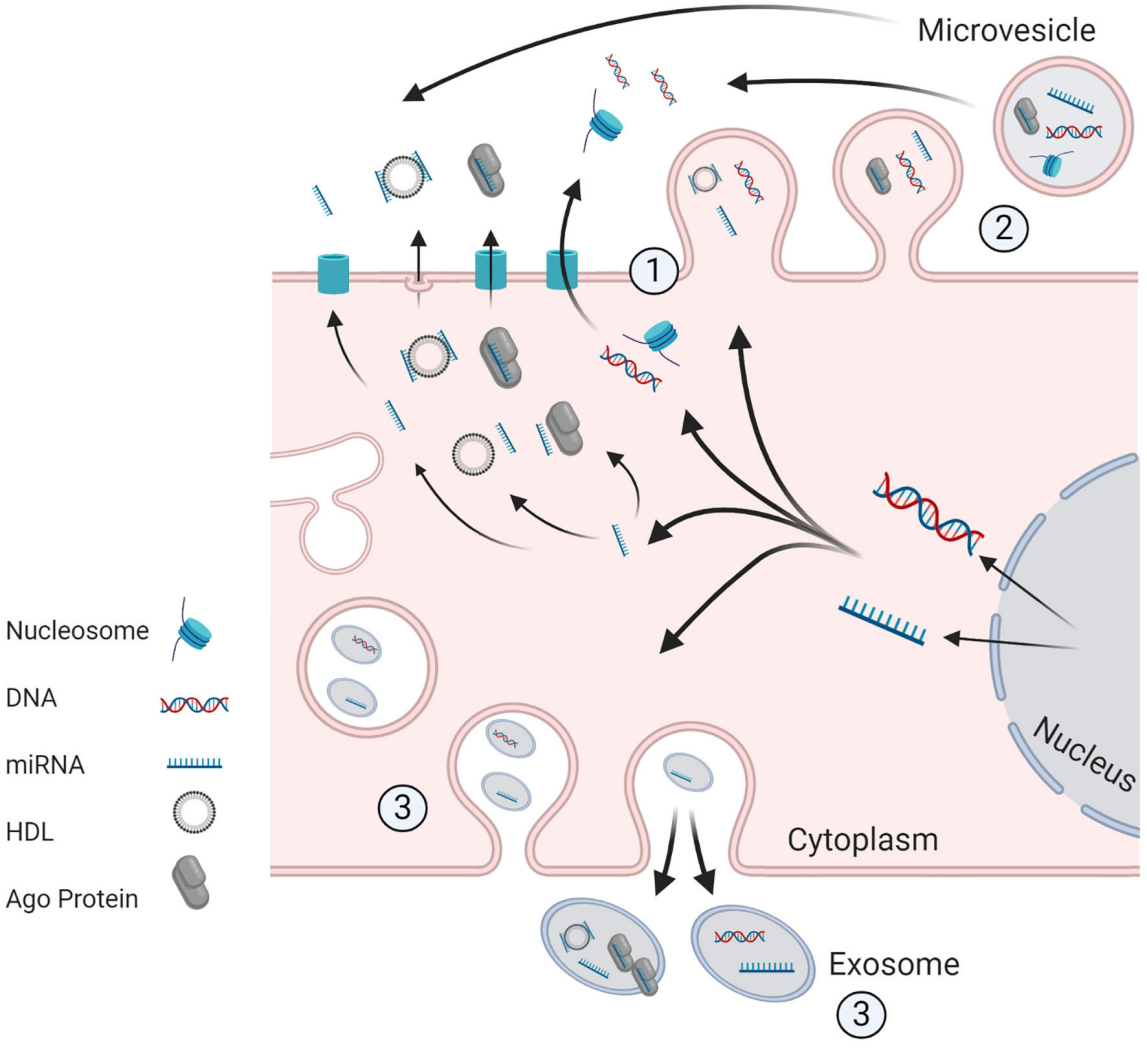
Potential Machanism of release of ctDNA and ct.miRNA from living tumor cells. (1) Active secretion, of unbound miRNA or in conjuction with Ago protein/HDL. DNA may be secreted in unbound from or in association with histones (as a nucleosome) or other proteins. The transporters for active secretion of nucleic acids are not fully characterized, however HDI/mRNA complexes are believed to be released through exocytosis. Alternative routes included secretion via formation of (2) microvesicles and (3) exosomes. Image created and exported form biorender.com under a paid subscription.

As genomic profiling becomes more cost-effective, biomarker discovery is increasingly moving away from non-specific tumor markers to highly refined “multi-omic” approaches. The aim of this review is to describe how knowledge of tumor biology and underlying molecular aberrations can be exploited for diagnostic, prognostic, and predictive biomarker design. We review emerging roles of circulating tumor cells, nucleic acids, messenger and microRNAs, and extracellular vesicles as tools for non-invasive diagnosis and surveillance in patients with or at risk of ovarian and/or endometrial cancer (Figure 3, Table 2).

**Table 2:** Definitions, advantages, and disadvantages of different Components of the liquid biopsy.

**Component**	**Abbreviation**	**Definition**	**Advantages**	**Disadvantages**
Circulating tumor Cells	CTCs	Free floating tumor cells in the blood circulation	Protein, gene expression, and genome analysis of tumor cells CTCs maybe cultured or used as xenografts for further functional study Reflection of tumor heterogeneity	• Rare • Difficult to extract and culture • Complexity of analysis due to Inter cellular heterogeneity
Circulating tumor DNA	ctDNA	Tumor derived DNA found in bodily fluids particularly plasma	Easy to extract and store Non-invasive method to analyse tumor genomics Tumor specific genomic alterations make detection relatively easy Evidence that ctDNA is representative of body wide tumor heterogeneity	Low concentration requiring high depth NGS
Circulating MicroRNAs	c.miRNA	RNA molecules typical 18–22 nucleotides in length, found in the circulation	Non-invasive	Rarely tumor specific Analysis relies on expression levels
Extracellular Vesicles	EVs	Lipid bilayer bound cell derived vesicles, 30–2,000 nm in diameter	Contain protein, lipids and nucleic acids derived from tumor Role in intracellular communication Role in cancer development	Heterogeneity of subtypes and composition Difficulty in extraction Difficulty in characterization
mRNA		Protein encoding mRNA found in the circulation	Ability to measure gene expression Tumor specific gene variants detectable	High level of fragmentation Difficult to amplify by NGS

## The “Liquid Biopsy”

The term “liquid biopsy” is a broad umbrella term used to describe a diverse array of tumor-derived material found in blood and other bodily fluids, including circulating tumors cells (CTCs), extracellular vesicles (EVs), and free nucleic acids. Since these factors are derived directly from tumor cells, they carry genetic, and other biological material from the tumors from which they are derived, including (in some cases) lipids and proteins, and in the case of CTCs, living cells. As they can be obtained from non-invasive produces, via repeated blood or fluid sampling, longitudinal monitoring is possible.

### Circulating Tumor Cells (CTCs)

Circulating tumors cells (CTCs) are tumor cells in the blood circulation that may exist as either single cells or cell conglomerates (clumps) (66). There is a building body of evidence to suggest that they are responsible for metastasis, although the cells must undergo a number of essential steps including intravasation into circulation, survival during dissemination, arrest at metastatic sites and then extravasation and colonization of the site before overt metastases can develop (67, 68). CTCs can be detected in low numbers even in early non-metastatic cancer indicating that dissemination of tumor cells in an early event. However, despite this early evidence of dissemination, few of these early cancers develop overt fmetastases and there is evidence that later disseminating CTCs may be more suited to the development of metastases (69). The development of metastasis is a complex process with many other factors required to aid metastasis beyond the presence of CTCs (69, 70), as is clearly demonstrated by the fact that modern techniques for CTC isolation can detect many thousands of cells in the circulation of aggressive tumors, and despite this only a restricted number of metastases develop in the majority of patients (66).

Factors limiting the use of routine analysis of CTCs in clinical practice is the scarce number of cells present within the blood even in patients with metastatic disease (often <1 CTC per ml of blood) (71). Analysis of CTCs is expensive, and technically challenging. Various technologies are available which detect CTCs through recognition of cell size, or expression of cell-surface antigens (72–74). Due to their rarity in blood, complex technology is needed to isolate and analyse CTCs, such as the epitope-dependent CellSearch® platform, and high-resolution imaging and analysis of dielectrophoretic movement of fluorochrome-labeled cells in the DEPArry™ microfluidic system (71, 75, 76). Furthermore, given that tumors are heterogeneous, containing multiple cell subpopulations, analysis of single CTCs may not be representative of whole tumor biology (77).

CTCs are a valuable liquid biopsy because they are living cells derived from tumors. This allows a multitude of potential investigations including functional analysis of cultured CTCs either directly or through organoid/xenograft studies (67). Molecular characterization is also possible including but not restricted to genome, gene expression, and protein analysis, most of which can now be on a single cell basis if required (75, 76, 78). Whole genome analysis of individual CTCs has shown that CTCs show a remarkable degree of variability, which gives valuable insight into the heterogeneity of the tumors from which they are derived (79). It is apparent that this degree of heterogeneity may not be captured by other liquid biopsy techniques (79). Recent developments in mRNA sequencing also allow genome-wide transcriptome analysis, allowing distinct gene expression patterns to be identified in individual CTCs (80). The analysis of CTC proteins is more difficult as there is no ability for amplification of these elements as there is for nucleic acids (via PCR), and cell staining can result in destruction of fragile cells. However, novel approaches using antibody barcode microarrays may allow protein characterization (81, 82) and the employment of miniscule nanowell chips may isolate single CTCs which can then be assayed for metabolic activity using fluorescent metabolic analogs (81) or for measurement of pH fluctuation (79).

The functional and molecular characterization of CTCs has prognostic significance and may provide the ability to monitor the development of cancer and response to anti-cancer therapy [(83, 84), #194]. The role of CTC analysis for screening, prognostication, and disease monitoring has been explored in a host of cancers, most notably breast, colorectal, prostate and lung (85–89). There have been a number of studies that have demonstrated the prognostic significance of CTCs in patients with ovarian cancer, with those patients with detectable CTCs having shorter overall survival and earlier relapse at all stages of disease (90, 91). In apparently optimally debulked patients, CTCs have been shown to be detectable in a subset of patients. These CTCs represent the presence of minimal residual disease even after R0 resection, and indicated a higher risk of death from disease (92). Presence of CTCs has also been shown to be superior than CA125 alone in the diagnosis of OC, where HE4-expression was used for CTC characterization alongside traditional CTC markers such as EpCam and cytokeratins (93). These results suggest the future inclusion of CTCs in diagnostic screening tools maybe helpful, if the utility can be proven in larger appropriately powered studies. However, there are still significant barrier to the establishment of CTCs as a reliable biomarker for OC, in particular an agreement on the exact surface markers used to isolate the CTCs, which may account for the variability in research findings.

CTCs have likewise been identified in patients with endometrial cancer (94) and have been shown to be associated with higher risk disease (95). Molecular profiling of CTCs has revealed a suite of genes that show differential regulation that may be subject to targeted therapy during future drug development trials (68). That CTCs are generally more common in aggressive disease suggests they may play an important role in understanding the biology of aggressive disease through further molecular characterization of cultured CTCs.

### Circulating Nucleic Acids

#### Circulating Tumor DNA (ctDNA)

Detection of circulating cell free DNA (cfDNA) in the plasma was first recorded in 1948 but it was not until 30 years later that levels were found to be elevated in cancer patients (96). Detection of tumor specific mutations in cfDNA was first identified in 1994 (97) and showed that the detected cfDNA was of tumor origin. This tumor derived fraction of cfDNA is termed circulating tumor DNA (ctDNA) and has been most extensively studied as a blood-borne (plasma) biomarker. However, it has also been described in a range of fluids including, urine, saliva, cerebrospinal fluid, and ascites (98).

The method by which cfDNA and, by extension, ctDNA is released into the blood and bodily fluids remains contentious. Some studies have suggested that necrosis and apoptosis are the main sources of ctDNA (99) but active secretion is also a plausible mechanism (100, 101). It should be noted that the majority of ctDNA is not likely to be derived from CTCs, as CTCs are rare in the circulation, and ctDNA levels are often 100–1,000 times the concentration that could be generated from apoptosis of CTCs alone (98). Independent of this mechanism ctDNA has been shown to be present in the majority of patients with advanced/metastatic cancer (102), and in many cancers at an early stage (103). It is simple to extract and once extracted can be stored for prolonged periods before analysis (104). In addition the detection of tumor specific alterations can allow detection of ctDNA at very low levels using digital droplet PCR (ddPCR) (105) or NGS incorporating unique molecular barcodes (106).

In cases where high sensitivity sequencing is needed, synthetic DNA sequences corresponding to a unique “barcode” are ligated to a DNA molecule (91). This is then followed by an amplification (PCR) step to massively increase the amount of DNA available for sequencing and typically results in multiple identical DNA molecules coalescing together via a variety of methods including Bead and Flow-based techniques, to allow sequencing (107). The final sequencing step typical involves DNA amplification with detection of nucleotide addition to the end of the amplifying DNA molecule (sequencing by synthesis) via release of photons (e.g., illumina) or protons (e.g., IonTorrent), or via the binding of labeled probes (e.g., Nanostring) (108). DNA sequences are recorded, and each base calledgiven a quality flag according to the strength of the signal for each base. Algorithms then allow this data to be improved, by checking all sequences that contain the marker of the original “barcoded” DNA molecule, and allow the determination of a consensus sequence for each original piece of DNA. Various algorithms also exist to filter out artifacts caused by the sequencing process (108, 109). The consensus DNA sequences can then be compared to the human genome sequence and variants from the standard sequence flagged. In the case of tumor DNA analysis, these flagged variants represent potential tumor specific variants. Though there is an ever increasing range of NGS techniques the majority of technologies use a variation of this description, and all nucleic acids can be detected using modifications of these sequencing techniques. The overall quality of the sequencing data produced is heavily reliant on the quality of the input DNA (110), and as such the isolation methods for each modality of the “liquid biopsy” can be very important.

Analysis of ctDNA has shown that ctDNA may be more representative of tumor heterogeneity than single tumor biopsies, as it is believed that all cells contribute to the pool of ctDNA (98). This allows assessment of body-wide tumor genomics with a single blood draw, which can be repeated at regular intervals via fresh blood sampling as required, allowing tumor monitoring (111). In addition the presence of ctDNA post operatively has been shown to predict tumor relapse indicating the detection of minimal residual disease (112–114). The presence of tumor specific mutations has significant promise in the direction of specific anti-cancer therapies (115, 116).

The main disadvantage of ctDNA is the high depth of sequencing required to detect low level ctDNA, particularly in early stage cancers, due to dilution by cfDNA derived from normal healthy cells. In addition, DNA analysis only represents a proportion of tumor biology, the remainder of which can only be assessed by tumor biopsy (eg., histology) or the assessment of other blood borne liquid biopsy methods. Raised plasma cfDNA levels have been detected in patients with endometrial or ovarian cancers compared to unaffected individuals (117).

Given that *TP53* is the most commonly altered gene in HGSOC, the detection of *TP53* variation in plasma cfDNA has been shown using both *TP53* specific ddPCR assays (118) and NGS sequencing approaches in blood and peritoneal fluid (119). This suggests that stratification of any new drugs targeted at p53 malfunction could be selected via interrogation of cfDNA, negating the need for invasive biopsies. *TP53* variant allele fraction (VAF) in cfDNA correlates with disease burden in patients with relapsed HGSOC. In addition, a *TP53* VAF decrease of ≤60% in response to chemotherapy has been shown to be a strong predictor of a short time to progression in patients (<6months) (120). However, ctDNA analysis is dynamic—if malignant ascites is drained prior to blood sampling, mean *TP53* VAF may be reduced. Furthermore, other factors such as exercise or inflammation may influence ctDNA levels.

Chromosomal instability in cfDNA has been proposed as a highly specific marker of malignancy in patients with adnexal masses, with improved performance compared to traditional biomarkers CA125 and RMI (121).

In comparison to ovarian cancer, there is a scarcity of published research supporting the utility of cfDNA analysis in patients with endometrial cancer, although elevated levels of plasma cfDNA have been recorded in affected patients (117). Unlike HGSOC, in which *TP53* mutations are ubiquitous, endometrial cancer lacks a single highly mutated gene that can be analyzed in cfDNA. However, the presence of ctDNA has been demonstrated by looking at tumor specific mutations in genes such as *KRAS* (122) or tumor whole exome sequencing followed by mutation specific ddPCR in specific subgroups (high grade serous) (123). The majority of endometrioid endometrial carcinomas have at least one driver mutation in one of four genes (*CTNNB1, KRAS, PTEN*, or *PIK3CA*). Paired tumor/plasma cfDNA NGS, targeting mutation hotspots in these four genes have been shown to detect tumor specific variants in 15/48 (31%) patients with early stage endometrial cancer, with bloods taken at the time of hysterectomy (124).

Elucidation of treatment-resistance mechanisms is challenging in all cancers, in part because of a paucity of available post-treatment tumor samples for analysis. The ability of cfDNA to reflect the body-wide tumor heterogeneity is therefore particularly useful when studying resistance mechanisms. Tumors caused by *BRCA1*/*BRCA2* pathogenic variants demonstrate increased sensitivity to platinum-based chemotherapy agents. BRCA-deficient tumors have defective homologous-recombination repair (HR), which results in impaired ability to repair the double-stranded DNA breaks caused by the alkylating action of platinum-based chemotherapeutics. As a result of defective HR, BRCA-deficient cells are dependent upon single-stranded DNA repair mechanisms, for example that facilitated by Poly ADP-ribose Polymerase (PARP)—making them particularly sensitive to synthetic lethality induced by PARP inhibition (125). Although patients with germline pathogenic variants in *BRCA1*/*BRCA2* demonstrate favorable response to both platinum-containing chemotherapy and PARP inhibitors, a large proportion of these patients go on to develop resistance to these therapies. A number of resistance mechanisms have been postulated, including the acquisition of secondary reversion mutations at or close to the initial loss-of-function variant, acting to restore functional protein production (126). These variants restore HR DNA repair, thereby removing the synthetic lethality of PARP inhibitors and allowing repair of DNA damage caused by the alkylating effect of platinum therapy.

A number of recent studies have shown that *BRCA* reversion mutations can be readily detected in the cfDNA of patients with both germline (103, 115, 127) and somatic (115) BRCA-mutated HGSOC. Detection of these reversion events in cfDNA represents a non-invasive method of predicting resistance to platinum-based therapy, and both primary and acquired resistance to PARP inhibitors (103, 115, 127). In the largest trial, in eight patients in whom cfDNA reversion mutations were detected, concurrent, or imminent disease progression was noted, with a median time to progression of 3.4 months, where detection preceded progression (115). As such testing for cfDNA *BRCA* reversion mutations may allow a non-invasive method for selection of those patients unlikely to respond to PARP inhibitors, and by repeated sampling predict resistance to PARP inhibitors before significant disease progression has occurred. These ideas must be proved in appropriate translational clinical trials, but show that cfDNA analysis may be of significant utility in therapy stratification and prediction of treatment resistance.

### Early Detection

Early detection of cancers has been a specific goal for the development of the use of liquid biopsies. Overall the use of cfDNA analysis has been most studied as a route for detecting early cancers due to the comparative ease with which can be collected and the ability to detect at very low levels using modern sequencing (128). New technologies can be used to supplement, rather than replace, traditional biomarkers to facilitate earlier detection of cancer with improved sensitivity without decreasing specificity (129). This can be demonstrated by the CancerSEEK multi-analyte blood test, which combines the detection of a ctDNA mutation (the majority of which are tumor-specific) with presence of traditional biomarkers (Ca125, CEA, Ca19-9) and less widely used blood borne proteins, including hepatocyte growth factor (HGF), osteopontin (OPN), myeloperoxidase (MPO), and tissue inhibitor of metalloproteinases 1 (TIMP-1) to improve sensitivity (129).

Given that circulating nucleic acids and exosomes may be present in extracellular fluids, another approach to improving cancer detection has been analyse samples collected as part of the Papanicolaou (Pap) test. The role of this test in early detection of cervical cancer is well-established, but detection of ovarian/endometrial cancers by this means via histopathological analysis is not usually possible. However, diagnosis of other gynecological malignancies may be facilitated by analysis of samples collected during Pap smears for assessment of sequence variation and copy number. The PapSEEK test assesses the fluid obtained during Pap tests for 18 specific variants characteristic of ovarian/endometrial cancers, as well as aneuploidy. The sensitivity of the PapSEEK test can be improved by collecting intrauterine samples, and with contemporaneous analysis of ctDNA (130).

### MicroRNAS

MicroRNAs (miRNAs) were first described in *C. Elegans* in 1993 (131) and our knowledge of this class of molecules has rapidly grown since this time. MicroRNAs are a group of small non-coding RNA molecules, typical 18-22 nucleotides in length that influence gene expression by binding with cis-regulatory regions in target messenger RNA, resulting in post-transcription regulation, through destabilization of the mRNA molecule (131, 132). The miRNA-mRNA network is very complex, as a single miRNA may have many hundreds of mRNA targets, and mRNA targets may be regulated by many different miRNAs (133). The human genome contains >1000 genes encoding miRNAs although the function of many of these small RNA molecules is yet to be established (132).

Cell free miRNAs (cf.miRNAs) were detected initially in 2007. Like cfDNA, cf.miRNAs can exist in plasma and various bodily fluids and their mechanism of release is not fully understood. Potential mechanisms include release from microvesicles or active secretion in association with protein complexes such as Ago2 (134) or high-density lipoproteins (135). Aberration of miRNA gene expression is seen in the majority of cancers, and miRNAs have been shown to alter the expression of a multitude of different genes. These changes in gene expression have been shown to be associated with most areas of tumor development from tumor initiation to metastasis (136).

Cell free miRNAs are relatively easy to isolate via commercially available kits and have been shown to be remarkably stable molecules despite the presence of endogenous RNase activity (137). This allows sufficient levels of miRNAs to be isolated for high quality sequencing. NGS sequencing following a reverse transcriptase step is the most commonly used analytical method (138). The reasons for their stability include their association with protein complexes or their packaging within extracellular vesicles (139). They have been detected in a number of body fluids besides plasma, including urine and saliva (140). Numerous clinical uses for cf.miRNAs have been studied in cancer including early diagnosis, monitoring of disease (140), predicting response to treatment and disease prognosis (141). Limitations in their analysis include that they are not specific to cancers, in that most miRNAs exists in both tumor and normal tissue. Analysis must rely on the relative levels of the cf.miRNA expression, which be difficult if the relative amount of cf.miRNA released from the tumors is low, or in early cancers. In addition alterations in miRNA expression are frequently shared between different tumor types, such as breast and lung cancer, as the same processes are frequently dysregulated in cancer. This ultimately reduces the specificity of cf.miRNA alteration to specific cancers.

Because of the more recent discover of cf.miRNA, research is this field is still relatively exploratory. In the quest for improved circulating biomarkers cf.miRNA has been evaluated and various labs have shown that cf.miRNA analysis can discriminate healthy from affected individuals (139). In particular single miRNAs such as miR-205 and let7f have been shown to have high diagnostic accuracy for early stage ovarian cancer, and let7f has been shown to have prognostic value, with low levels indicating a poor prognosis (142).

Studies in endometrial cancer have also shown differential levels of cf.miRNA between healthy and affected patients. In one study miR-15b, miR-27a, and miR-223 were found to be differentially expressed between patients with endometrioid endometrial cancer and unaffected individual and therefore may have a role therefore in improving diagnosis of endometrial cancer pending further investigation (143).

Overall the work into cf.miRNAs is generating a huge number of potential new biomarkers (136, 141, 144), and significant work is needed to increase our understanding of these markers in order to improve our ability to utilize them.

### mRNA

Protein coding messenger RNA molecules (mRNA) are known to be present in plasma samples in addition to short-non-coding RNAs and other nucleic acids (138). Like cf.miRNAS, cell free mRNAS (cfmRNA) are thought to be found in association with extracellular vesicles, as well as free in the circulation (145). Analysis of cfmRNA, allows measurement of protein encoding gene expression and detection of tumor specific mutational variants, neither of which is possible using cf.miRNA. However, the latter is also possible by analysis of cfDNA which is more stable and requires less specialist extraction methods (145). Far fewer reports are available on the use of cfmRNA than cf.miRNA, because it is less stable (frequently degraded) and less abundant in the circulation, making it more difficult to detect and analyse (145).

### Extracellular Vesicles

Tumor derived extracellular vesicles (EVs) are lipid bilayer bound cell derived vesicles, 30–2,000 nm in diameter. Initially thought to be nothing more than membrane debris they have since been shown to have important roles in intracellular communication via the transfer of proteins, lipids and nucleic acids (146, 147). The three main classes are; exosomes, microvesicles and apoptotic bodies. Exosomes are derived from the endolysosomal pathway and microvesicles via budding from the plasma membrane. Apoptotic bodies are derived from the controlled death of cells via the similarly named apoptotic pathway. EVs derived from these different routes vary in size and composition, including enrichment for certain surface markers. However, the heterogeneity of different surface markers can allow for enrichment of these subtypes via marker selection.

EVs appear to have a diverse role including the regulation of immune responses, tissue repair and blood coagulation (146). Exosomes in particular play a central role in antigen presentation and immune surveillance and activation (148, 149), whilst both exosomes and microvesicles mediate genetic inter-cellular communication, by carrying nucleic acids between cells (150). Given their central role in regulating cellular processes it is not surprising that EVs may have an important role in pathogenesis of cancer and a number of other diseases. Their role in tumor development is diverse, including stimulation of growth and angiogenesis (151), promotion of immune escape and the formation of pre-metastatic niches (70).

Given that EVs appear to play a direct role in many fundamental steps of tumor initiation and progression there is growing interest in the modulation of their effect. This includes inhibition of their formation, extracellular release or uptake and blocking specific extracellular EV components (146). EVs contain many of the other circulating components including DNA, mRNA, microRNA, and other non-coding RNAs, making analysis of any one component difficult (150). Indeed they may be one of the sources of these components on plasma analysis, although it should be noted that the composition of circulating nucleic acids differs from those found in EVs, and that the different subtypes of EV also differ in their composition (149, 152).

Difficulties with EVs are inherent in their heterogeneity and complexity. Research results based on an EV analysis from a single cell type can differ dependent on cell culture conditions, differences in purification protocols or methods used to characterize the EV (153, 154). For example ultrafugation has been the gold standard for exosome purification but concern regarding low activity of those EVs collected has led to a range of newer techniques including liquid chromatography or marker precipitation based separation (146, 155).

Tumor cells are well-known to produce large amounts of EVs. Because of their role in cellular communication, their complexity in terms of composition and their varied role in tumor growth and developments EVs contain a large amount of biological information about the tumors from which they arise. They therefore have been evaluated for varied roles in cancer including prognostic biomarkers (156). Disease monitoring is possible from EVs in blood plasma and a range of other bodily fluids such as urine and saliva (151, 157). Of interest plasma EVs maybe able to differentiate affected and unaffected patients, and may distinguish between those with early and those with late disease. They may also be enriched for immunosuppressive proteins including PD-L1, PD-1 and CTLA4, and high PD-levels have correlated with high disease activity and advanced stage of disease. Finally, monitoring of protein levels in EVs has also been shown to correlate with disease response, suggesting EVs may play a role in monitoring of response to therapy. This suggests EVs can be used as a measure of disease progression and prediction of advanced disease, and may play a role in treatment selection in the future, e.g., through stratification for the use immune checkpoint inhibitors (155, 156). There is growing evidence that due to the readily accessible nature of EVs, and the bioactive molecules that they contain, EVs may also have a role in detection of early stage disease (158).

Difficulties with the use of EVs center on methods for their extraction and purification. However, the diversity of the potential uses for EVs also means that summarizing all the current research in this review is not possible.

As EVs contain a variety of different classes of molecule significant work has concentrated on the characterization of known molecules within them. For example exosomal miRNAs could serve as potential biomarkers (159) and soluble E-cadherin bound to exosomes has been identified as both a potential biomarker and therapeutic target (160) in OC.

Herrero et al. have recently demonstrated the utility of ExoGAG technology to enrich for EVs in patients with endometrial cancer, and to facilitate detection of two known prognostic and predictive endometrial cancer biomarkers; L1 cell adhesion molecule (L1CAM) and Annexin A2 (ANXA2) in the samples (155). Increased levels of ANAX2 in EVs were found to correlate with high-risk histology, grade, stage and risk of recurrence, suggesting it may play a role in disease monitoring, recurrence and detection of early disease. Further work into the use of ANAX2 in early detection of EC is required to elucidate the best timing of samples and confirm the cut-off levels above which disease is likely to be present This study demonstrates how known protein based biomarkers maybe of enhanced value following the analysis of EVs.

The study of EVs not only reveals markers that can be monitored during therapy but also targets for therapy as EVs are known to be involved in many elements of tumorigenesis or promote the development of metastases through enhancement of the premetastatic niche. For example exosomes may promote cell invasion through transfer of surface glycoproteins in ovarian cancer (161), and exosomes released from tumor associated macrophages transfer miRNAs that contribute to an immunosuppressive microenvironment (162). Therefore, by contributing to the understanding of ovarian cancer and other cancers this may yet translate to new targets for anticancer therapy and the eventual development of new targeted drugs.

## Summary

As described the “liquid biopsy” has a wide potential for improving our understanding of cancer, including measurement of both temporal and spatial heterogeneity both of which are substantial barriers to the development of personalized anti- cancer therapies. As such, use of the liquid biopsy promises assessment of tumor heterogeneity at a greater frequency and with reduced morbidity compared to tissue biopsies. Their development therefore promises to improve detection and monitoring of tumors and identification of targeted treatment guided by precision medicine. A critical factor in the reduction of the morbidity and mortality of endometrial cancer and ovarian cancer is improvement of these very issues. Therefore, we further discuss current developments in the field of precision oncology in endometrial cancer and ovarian cancer and how improving knowledge in “liquid biopsy” research are contributing to the development of both targeted anti-cancer therapy and disease monitoring.

## Author Contributions

MO and TM conceived of the review, reviewed the literature, and drafted and approved of the final manuscript. All authors contributed to the article and approved the submitted version.

## Conflict of Interest

TM has received speaking honoraria from Roche, Novartis, Astra Zeneca, MSD, and Merck. The remaining author declares that the research was conducted in the absence of any commercial or financial relationships that could be construed as a potential conflict of interest.

## References

[1] Bray F Ferlay J Soerjomataram I Siegel RL Torre LA Jemal A. Global cancer statistics 2018: GLOBOCAN estimates of incidence and mortality worldwide for 36 cancers in 185 countries. CA Cancer J Clin. (2018) 68:394–424. 10.3322/caac.21492 30207593

[2] Lodish H Berk A Zipursky SL. Proto-oncogenes and tumor suppressor genes. In: Freeman WH editor. Molecular Cell Biology. New York, NY: W. H. Freeman and Company (2000).

[3] Hanahan D Weinberg RA. Hallmarks of cancer: the next generation. Cell. (2011) 144:646–74. 10.1016/j.cell.2011.02.013 21376230

[4] Moore PS Chang Y. Why do viruses cause cancer? Highlights of the first century of human tumour virology. Nat Rev Cancer. (2010) 10:878–89. 10.1038/nrc2961 21102637 PMC3718018

[5] Stratton MR. Journeys into the genome of cancer cells. EMBO Mol Med. (2013) 5:169–72. 10.1002/emmm.201202388 23339072 PMC3569633

[6] Catenacci DV Amico AL Nielsen SM Geynisman DM Rambo B Carey GB. Tumor genome analysis includes germline genome: are we ready for surprises? Int J Cancer. (2015) 136:1559–67. 10.1002/ijc.29128 25123297 PMC4303936

[7] Mansfield EA. FDA perspective on companion diagnostics: an evolving paradigm. Clin Cancer Res. (2014) 20:1453–7. 10.1158/1078-0432.CCR-13-1954 24634468

[8] Gatius S Cuevas D Fernández C Roman-Canal B Adamoli V Piulats JM. Tumor heterogeneity in endometrial carcinoma: practical consequences. Pathobiology. (2018) 85:35–40. 10.1159/000475529 28614814

[9] Allott EH Geradts J Sun X Cohen SM Zirpoli GR Khoury T. Intratumoral heterogeneity as a source of discordance in breast cancer biomarker classification. Breast Cancer Res. (2016) 18:68–79. 10.1186/s13058-016-0725-1 27349894 PMC4924300

[10] Diaz LA Jr Williams RT Wu J Kinde I Hecht JR Berlin J. The molecular evolution of acquired resistance to targeted EGFR blockade in colorectal cancers. Nature. (2012) 486:537–40. 10.1038/nature11219 22722843 PMC3436069

[11] de Bruin EC McGranahan N Mitter R Salm M Wedge DC Yates L. Spatial and temporal diversity in genomic instability processes defines lung cancer evolution. Science. (2014) 346:251–6. 10.1126/science.1253462 25301630 PMC4636050

[12] Arbyn M Rebolj M De Kok IM Fender M Becker N O'Reilly M. The challenges of organising cervical screening programmes in the 15 old member states of the European Union. Eur J Cancer. (2009) 45:2671–8. 10.1016/j.ejca.2009.07.016 19695867

[13] Jansen EEL Zielonke N Gini A Anttila A Segnan N Vokó Z. Effect of organised cervical cancer screening on cervical cancer mortality in Europe: a systematic review. Eur J Cancer. (2020) 127:207–23. 10.1016/j.ejca.2019.12.013 31980322

[14] Drolet M Bénard É Pérez N Brisson M. Population-level impact and herd effects following the introduction of human papillomavirus vaccination programmes: updated systematic review and meta-analysis. Lancet. (2019) 394:497–509. 10.1016/S0140-6736(19)30298-3 31255301 PMC7316527

[15] World Health Organisation. A Global Strategy for Elimination of Cervical Cancer. (2020). Available online at: https://www.who.int/activities/a-global-strategy-for-elimination-of-cervical-cancer

[16] Brisson M Kim JJ Canfell K Drolet M Gingras G Burger EA. Impact of HPV vaccination and cervical screening on cervical cancer elimination: a comparative modelling analysis in 78 low-income and lower-middle-income countries. Lancet. (2020) 395:575–90. 10.1016/S0140-6736(20)30068-4 32007141 PMC7043009

[17] Gelincik O Ibrahim H Ozkan M Ahadova A Sei S Shoemaker R. Abstract 2732: Frameshift neoantigen vaccination prevent Lynch syndrome mouse model intestinal cancer. Cancer Res. (2019) 79(13 Suppl):2732. 10.1158/1538-7445.AM2019-2732

[18] Majumder S Shah R Elias J Manoharan M Shah P Kumari A. A cancer vaccine approach for personalized treatment of Lynch Syndrome. Sci Rep. (2018) 8:12122. 10.1038/s41598-018-30466-x 30108227 PMC6092430

[19] von Knebel Doeberitz M Kloor M. Towards a vaccine to prevent cancer in Lynch syndrome patients. Fam Cancer. (2013) 12:307–12. 10.1007/s10689-013-9662-7 23760517

[20] Constantine GD Kessler G Graham S Goldstein SR. Increased incidence of endometrial cancer following the women's health initiative: an assessment of risk factors. J Womens Health. (2019) 28:237–43. 10.1089/jwh.2018.6956 30484734 PMC6390656

[21] Lortet-Tieulent J Ferlay J Bray F Jemal A. International patterns and trends in endometrial cancer incidence, 1978-2013. J Natl Cancer Inst. (2018) 110:354–61. 10.1093/jnci/djx214 29045681

[22] Jasen P. From the “silent killer” to the “whispering disease”: ovarian cancer and the uses of metaphor. Med Hist. (2009) 53:489–512. 10.1017/S0025727300000521 19876511 PMC2766137

[23] Kuchenbaecker KB Hopper JL Barnes DR Phillips KA Mooij TM Roos-Blom MJ. Risks of breast, ovarian, and contralateral breast cancer for BRCA1 and BRCA2 mutation carriers. JAMA. (2017) 317:2402–16. 10.1001/jama.2017.7112 28632866

[24] Yang X Song H Leslie G Engel C Hahnen E Auber B. Ovarian and breast cancer risks associated with pathogenic variants in RAD51C and RAD51D. J Natl Cancer Inst. (2020) 28:djaa030. 10.1093/jnci/djaa030 32107557 PMC7735771

[25] Ramus SJ Song H Dicks E Tyrer JP Rosenthal AN Intermaggio MP. Germline mutations in the BRIP1, BARD1, PALB2, and NBN genes in women with ovarian cancer. J Natl Cancer Inst. (2015) 107:djv214. 10.1093/jnci/djv214 26315354 PMC4643629

[26] Yang X Leslie G Doroszuk A Schneider S Allen J Decker B. Cancer risks associated with germline PALB2 pathogenic variants: an international study of 524 families. J Clin Oncol. (2020) 38:674–85. 10.1200/JCO.19.01907 31841383 PMC7049229

[27] Garg K Karnezis AN Rabban JT. Uncommon hereditary gynaecological tumour syndromes: pathological features in tumours that may predict risk for a germline mutation. Pathology. (2018) 50:238–56. 10.1016/j.pathol.2017.10.009 29373116

[28] Møller P Seppälä TT Bernstein I Holinski-Feder E Sala P Gareth Evans D. Cancer risk and survival in path_MMR carriers by gene and gender up to 75 years of age: a report from the prospective lynch syndrome database. Gut. (2018) 67:1306–16. 10.1136/gutjnl-2017-314057 28754778 PMC6031262

[29] Ten Broeke SW van der Klift HM Tops CMJ Aretz S Bernstein I. Cancer risks for PMS2-associated lynch syndrome. J Clin Oncol. (2018) 36:2961–8. 10.1200/JCO.2018.78.4777 30161022 PMC6349460

[30] Tan MH Mester JL Ngeow J Rybicki LA Orloff MS Eng C. Lifetime cancer risks in individuals with germline PTEN mutations. Clin Cancer Res. (2012) 18:400–7. 10.1158/1078-0432.CCR-11-2283 22252256 PMC3261579

[31] Bellido F Pineda M Aiza G Valdés-Mas R Navarro M Puente DA. POLE and POLD1 mutations in 529 kindred with familial colorectal cancer and/or polyposis: review of reported cases and recommendations for genetic testing and surveillance. Genet Med. (2016) 18:325–32. 10.1038/gim.2015.75 26133394 PMC4823640

[32] Lakhani SR Manek S Penault-Llorca F Flanagan A Arnout L Merrett S. Pathology of ovarian cancers in BRCA1 and BRCA2 carriers. Clin Cancer Res. (2004) 10:2473–81. 10.1158/1078-0432.CCR-1029-3 15073127

[33] Wu M Krishnamurthy K. Peutz-Jeghers Syndrome, in StatPearls [Internet]. Treasure Island, FL: StatPearls Publishing (2019). 30570978

[34] Fuller PJ Leung D Chu S. Genetics and genomics of ovarian sex cord-stromal tumors. Clin Genet. (2017) 91:285–91. 10.1111/cge.12917 27813081

[35] Ferry JA Young RH Engel G Scully RE. Oxyphilic sertoli cell tumor of the ovary: a report of three cases, two in patients with the peutz-jeghers syndrome. Int J Gynecol Pathol. (1994) 13:259–66. 10.1097/00004347-199407000-00010 7523322

[36] Nakamura K Banno K Yanokura M Iida M Adachi M Masuda K. Features of ovarian cancer in Lynch syndrome (Review). Mol Clin Oncol. (2014) 2:909–16. 10.3892/mco.2014.397 25279173 PMC4179837

[37] Meyer LA Broaddus RR Lu KH. Endometrial cancer and Lynch syndrome: clinical and pathologic considerations. Cancer Control. (2009) 16:14–22. 10.1177/107327480901600103 19078925 PMC3693757

[38] Wong A Ngeow J. Hereditary syndromes manifesting as endometrial carcinoma: how can pathological features aid risk assessment? Biomed Res Int. (2015) 2015:219012. 10.1155/2015/219012 26161390 PMC4486295

[39] Kotsopoulos J Gronwald J Karlan B Rosen B Huzarski T Moller P. Age-specific ovarian cancer risks among women with a BRCA1 or BRCA2 mutation. Gynecol Oncol. (2018) 150:85–91. 10.1016/j.ygyno.2018.05.011 29793803

[40] Vasen HF Blanco I Aktan-Collan K Gopie JP Alonso A Aretz S. Revised guidelines for the clinical management of lynch syndrome (HNPCC): recommendations by a group of European experts. Gut. (2013) 62:812–23. 10.1136/gutjnl-2012-304356 23408351 PMC3647358

[41] Crosbie EJ Ryan NAJ Arends MJ Bosse T Burn J Cornes JM. The manchester international consensus group recommendations for the management of gynecological cancers in lynch syndrome. Genet Med. (2019) 21:2390–400. 10.1038/s41436-019-0489-y 30918358 PMC6774998

[42] Gaba F Manchanda R. Systematic review of acceptability, cardiovascular, neurological, bone health and HRT outcomes following risk reducing surgery in BRCA carriers. Best Pract Res Clin Obstet Gynaecol. (2020) 65:46–65. 10.1016/j.bpobgyn.2020.01.006 32192936

[43] Kyo S Ishikawa N Nakamura K Nakayama K. The fallopian tube as origin of ovarian cancer: change of diagnostic and preventive strategies. Cancer Med. (2020) 9:421–31. 10.1002/cam4.2725 31769234 PMC6970023

[44] Gaba F Piek J Menon U Manchanda R. Risk-reducing early salpingectomy and delayed oophorectomy as a two-staged alternative for primary prevention of ovarian cancer in women at increased risk: a commentary. BJOG. (2019) 126:831–9. 10.1111/1471-0528.15651 30735593

[45] Clancy C Burke JP Chang KH Coffey JC. The effect of hysterectomy on colonoscopy completion: a systematic review and meta-analysis. Dis Colon Rectum. (2014) 57:1317–23. 10.1097/DCR.0000000000000223 25285700

[46] Paraskevaidi M Morais CLM Ashton KM Stringfellow HF McVey RJ Ryan NAJ. Detecting endometrial cancer by blood spectroscopy: a diagnostic cross-sectional study. Cancers. (2020) 12:1256. 10.3390/cancers12051256 32429365 PMC7281323

[47] Rosenthal AN Fraser LSM Philpott S Manchanda R Burnell M Badman P. Evidence of stage shift in women diagnosed with ovarian cancer during phase ii of the united kingdom familial ovarian cancer screening study. J Clin Oncol. (2017) 35:1411–20. 10.1200/JCO.2016.69.9330 28240969 PMC5455461

[48] Jacobs I Oram D Fairbanks J Turner J Frost C Grudzinskas JG. A risk of malignancy index incorporating CA 125, ultrasound and menopausal status for the accurate preoperative diagnosis of ovarian cancer. Br J Obstet Gynaecol. (1990) 97:922–9. 10.1111/j.1471-0528.1990.tb02448.x 2223684

[49] Moss EL Hollingworth J Reynolds TM. The role of CA125 in clinical practice. J Clin Pathol. (2005) 58:308–12. 10.1136/jcp.2004.018077 15735166 PMC1770590

[50] Marcus CS Maxwell GL Darcy KM Hamilton CA McGuire WP. Current approaches and challenges in managing and monitoring treatment response in ovarian cancer. J Cancer. (2014) 5:25–30. 10.7150/jca.7810 24396495 PMC3881218

[51] Rustin GJ van der Burg ME Griffin CL Guthrie D Lamont A. Early versus delayed treatment of relapsed ovarian cancer (MRC OV05/EORTC 55955): a randomised trial. Lancet. (2010) 376:1155–63. 10.1016/S0140-6736(10)61268-8 20888993

[52] Boylan KLM Geschwind K Koopmeiners JS Geller MA Starr TK Skubitz APN. A multiplex platform for the identification of ovarian cancer biomarkers. Clin Proteomics. (2017) 14:34. 10.1186/s12014-017-9169-6 29051715 PMC5634875

[53] Whitwell HJ Worthington J Blyuss O Gentry-Maharaj A Ryan A Gunu R. Improved early detection of ovarian cancer using longitudinal multimarker models. Br J Cancer. (2020) 122:847–56. 10.1038/s41416-019-0718-9 31937926 PMC7078315

[54] The Cancer Genome Atlas Research Network. Integrated genomic analyses of ovarian carcinoma. Nature. (2011) 474:609–15. 10.1038/nature10166 21720365 PMC3163504

[55] Moschetta M George A Kaye SB Banerjee S. BRCA somatic mutations and epigenetic BRCA modifications in serous ovarian cancer. Ann Oncol. (2016) 27:1449–55. 10.1093/annonc/mdw142 27037296

[56] Hoppe MM Sundar R Tan DSP Jeyasekharan AD. Biomarkers for homologous recombination deficiency in cancer. J Natl Cancer Inst. (2018) 110:704–13. 10.1093/jnci/djy085 29788099

[57] The Cancer Genome Atlas Research Network Levine DA. Integrated genomic characterization of endometrial carcinoma. Nature. (2013) 497:67–73. 10.1038/nature12113 23636398 PMC3704730

[58] Le DT Durham JN Smith KN Wang H Bartlett BR Aulakh LK. Mismatch repair deficiency predicts response of solid tumors to PD-1 blockade. Science. (2017) 357:409–13. 10.1126/science.aan6733 28596308 PMC5576142

[59] Pal T Permuth-Wey J Kumar A Sellers TA. Systematic review and meta-analysis of ovarian cancers: estimation of microsatellite-high frequency and characterization of mismatch repair deficient tumor histology. Clin Cancer Res. (2008) 14:6847–54. 10.1158/1078-0432.CCR-08-1387 18980979 PMC2655731

[60] Murphy MA Wentzensen N. Frequency of mismatch repair deficiency in ovarian cancer: a systematic review. Int J Cancer. (2011) 129:1914–22. 10.1002/ijc.25835 21140452 PMC3107885

[61] Ryan NAJ McMahon R Tobi S Snowsill T Esquibel S Wallace AJ. The proportion of endometrial tumours associated with Lynch syndrome (PETALS): a prospective cross-sectional study. PLoS Med. (2020) 17:e1003263. 10.1371/journal.pmed.1003263 32941469 PMC7497985

[62] Lee V Murphy A Le DT Diaz LA Jr. mismatch repair deficiency and response to immune checkpoint blockade. Oncologist. (2016) 21:1200–11. 10.1634/theoncologist.2016-0046 27412392 PMC5061538

[63] Bartl T Paspalj V Polterauer S Grimm C. Current state and perspectives of checkpoint inhibitors in ovarian cancer treatment. Magaz Eur Med Oncol. (2020) 13:202–6. 10.1007/s12254-020-00579-z

[64] Green AK Feinberg J Makker V. A review of immune checkpoint blockade therapy in endometrial cancer. Am Soc Clin Oncol Educ Book. (2020) 40:1–7. 10.1200/EDBK_280503 32213091

[65] Kandalaft LE Odunsi K Coukos G. Immune therapy opportunities in ovarian cancer. Am Soc Clin Oncol Educ Book. (2020) 40:1–13. 10.1200/EDBK_280539 32412818

[66] Allard WJ Matera J Miller MC Repollet M Connelly MC Rao C. Tumor cells circulate in the peripheral blood of all major carcinomas but not in healthy subjects or patients with nonmalignant diseases. Clin Cancer Res. (2004) 10:6897–904. 10.1158/1078-0432.CCR-04-0378 15501967

[67] Pantel K Speicher MR. The biology of circulating tumor cells. Oncogene. (2016) 35:1216–24. 10.1038/onc.2015.192 26050619

[68] Alonso-Alconada L Muinelo-Romay L Madissoo K Diaz-Lopez A Krakstad C Trovik J. Molecular profiling of circulating tumor cells links plasticity to the metastatic process in endometrial cancer. Mol Cancer. (2014) 13:223. 10.1186/1476-4598-13-223 25261936 PMC4190574

[69] Vanharanta S Massague J. Origins of metastatic traits. Cancer Cell. (2013) 24:410–21. 10.1016/j.ccr.2013.09.007 24135279 PMC3998120

[70] Peinado H Zhang H Matei IR Costa-Silva B Hoshino A Rodrigues G. Pre-metastatic niches: organ-specific homes for metastases. Nat Rev Cancer. (2017) 17:302–17. 10.1038/nrc.2017.6 28303905

[71] Alvarez Cubero MJ Lorente JA Robles-Fernandez I Rodriguez-Martinez A Puche JL Serrano MJ. Circulating tumor cells: markers and methodologies for enrichment and detection. Methods Mol Biol. (2017) 1634:283–303. 10.1007/978-1-4939-7144-2_24 28819860

[72] Alix-Panabières C Pantel K. Challenges in circulating tumour cell research. Nat Rev Cancer. (2014) 14:623–31. 10.1038/nrc3820 25154812

[73] Alix-Panabières C Pantel K. Clinical applications of circulating tumor cells and circulating tumor dna as liquid biopsy. Cancer Discov. (2016) 6:479–91. 10.1158/2159-8290.CD-15-1483 26969689

[74] Li N Zuo H Chen L Liu H Zhou J Yao Y. Circulating tumor cell detection in epithelial ovarian cancer using dual-component antibodies targeting EpCAM And FRα. Cancer Manage Res. (2019) 11:10939–948. 10.2147/CMAR.S211455 32021417 PMC6978676

[75] Shaw JA Guttery DS Hills A Fernandez-Garcia D Page K Rosales BM. Mutation analysis of cell-free DNA and single circulating tumor cells in metastatic breast cancer patients with high CTC counts. Clin Cancer Res. (2017) 23:88–96. 10.1158/1078-0432.CCR-16-0825 27334837 PMC6241844

[76] Blassl C Kuhlmann JD Webers A Wimberger P Fehm T Neubauer H. Gene expression profiling of single circulating tumor cells in ovarian cancer - establishment of a multi-marker gene panel. Mol Oncol. (2016) 10:1030–42. 10.1016/j.molonc.2016.04.002 27157930 PMC5423187

[77] Barriere G Fici P Gallerani G Fabbri F Zoli W Rigaud M. Circulating tumor cells and epithelial, mesenchymal and stemness markers: characterization of cell subpopulations. Ann Transl Med. (2014) 2:109. 10.3978/j.issn.2305-5839.2014.10.04 25489583 PMC4245517

[78] Huang L Ma F Chapman A Lu S Xie XS. Single-cell whole-genome amplification and sequencing: methodology and applications. Annu Rev Genomics Hum Genet. (2015) 16:79–102. 10.1146/annurev-genom-090413-025352 26077818

[79] Keller L Pantel K. Unravelling tumour heterogeneity by single-cell profiling of circulating tumour cells. Nat Rev Cancer. (2019) 19:553–67. 10.1038/s41568-019-0180-2 31455893

[80] Ramsköld D Luo S Wang YC Li R Deng Q Faridani OR. Full-length mRNA-Seq from single-cell levels of RNA and individual circulating tumor cells. Nat Biotechnol. (2012) 30:777–82. 10.1038/nbt.2282 22820318 PMC3467340

[81] Zhang Y Tang Y Sun S Wang Z Wu W Zhao X. Single-cell codetection of metabolic activity, intracellular functional proteins, and genetic mutations from rare circulating tumor cells. Anal Chem. (2015) 87:9761–8. 10.1021/acs.analchem.5b01901 26378744

[82] O'Shannessy DJ Davis DW Anderes K Somers EB. Isolation of circulating tumor cells from multiple epithelial cancers with apostream(®) for detecting (or monitoring) the expression of folate receptor alpha. Biomarker insights. (2016) 11:7–18. 10.4137/BMI.S35075 26848256 PMC4737520

[83] Asante D.-B Calapre L Ziman M Meniawy TM Gray ES. Liquid biopsy in ovarian cancer using circulating tumor DNA and cells: ready for prime time? Cancer Letters. (2020) 468:59–71. 10.1016/j.canlet.2019.10.014 31610267

[84] Banys-Paluchowski M Fehm T Neubauer H Paluchowski P Krawczyk N Meier-Stiegen F. Clinical relevance of circulating tumor cells in ovarian, fallopian tube and peritoneal cancer. Arch Gynecol Obstet. (2020). 301:1027–35. 10.1007/s00404-020-05477-7 32144573 PMC7103005

[85] Leroy S Benzaquen J Mazzetta A Marchand-Adam S Padovani B Israel-Biet D. Circulating tumour cells as a potential screening tool for lung cancer (the AIR study): protocol of a prospective multicentre cohort study in France. BMJ Open. (2017) 7:e018884. 10.1136/bmjopen-2017-018884 29282271 PMC5770962

[86] Ilie M Szafer-Glusman E Hofman V Chamorey E Lalvee S Selva E. Detection of PD-L1 in circulating tumor cells and white blood cells from patients with advanced non-small-cell lung cancer. Ann Oncol. (2018) 29:193–9. 10.1093/annonc/mdx636 29361135

[87] Mesquita B Rothwell DG Burt DJ Chemi F Fernandez-Gutierrez F Slane-Tan D. Molecular analysis of single circulating tumour cells following long-term storage of clinical samples. Mol Oncol. (2017) 11:1687–97. 10.1002/1878-0261.12113 28741788 PMC5709616

[88] Cabel L Proudhon C Gortais H Loirat D Coussy F Pierga JY. Circulating tumor cells: clinical validity and utility. Int J Clin Oncol. (2017) 22:421–30. 10.1007/s10147-017-1105-2 28238187

[89] Sumanasuriya S Omlin A Armstrong A Attard G Chi KN Bevan CL. Consensus statement on circulating biomarkers for advanced prostate cancer. Eur Urol Oncol. (2018) 1:151–9. 10.1016/j.euo.2018.02.009 31100240

[90] Poveda A Kaye SB McCormack R Wang S Parekh T Ricci D. Circulating tumor cells predict progression free survival and overall survival in patients with relapsed/recurrent advanced ovarian cancer. Gynecol Oncol. (2011) 122:567–72. 10.1016/j.ygyno.2011.05.028 21664658

[91] Zeng L Liang X Liu Q Yang Z. The predictive value of circulating tumor cells in ovarian cancer: a meta analysis. Int J Gynecol Cancer. (2017) 27:1109–17. 10.1097/IGC.0000000000000459 25893279

[92] Obermayr E Bednarz-Knoll N Orsetti B Weier HU Lambrechts S Castillo-Tong DC. Circulating tumor cells: potential markers of minimal residual disease in ovarian cancer? a study of the OVCAD consortium. Oncotarget. (2017) 8:106415–28. 10.18632/oncotarget.22468 29290959 PMC5739744

[93] Guo YX Neoh KH Chang XH Sun Y Cheng HY Ye X. Diagnostic value of HE4+ circulating tumor cells in patients with suspicious ovarian cancer. Oncotarget. (2018) 9:7522–33. 10.18632/oncotarget.23943 29484129 PMC5800921

[94] Bogani G Liu MC Dowdy SC Cliby WA Kerr SE Kalli KR. Detection of circulating tumor cells in high-risk endometrial cancer. Anticancer Res. (2015) 35:683−7. Available online at: http://ar.iiarjournals.org/content/35/2/683.long 25667446

[95] Kiss I Kolostova K Matkowski R Jedryka M Czekanski A Pavlasek J. Correlation between disease stage and the presence of viable circulating tumor cells in endometrial cancer. Anticancer Res. (2018) 38:2983–987. 10.21873/anticanres.12550 29715128

[96] Leon SA Shapiro B Sklaroff DM Yaros MJ. Free DNA in the serum of cancer patients and the effect of therapy. Cancer Res. (1977) 37:646–50. 837366

[97] Sorenson GD Pribish DM Valone FH Memoli VA Bzik DJ Yao SL. Soluble normal and mutated DNA sequences from single-copy genes in human blood. Cancer Epidemiol Biomarkers Prev. (1994) 3:67–71. 8118388

[98] Thierry AR El Messaoudi S Gahan PB Anker P Stroun M. Origins, structures, and functions of circulating DNA in oncology. Cancer Metastasis Rev. (2016) 35:347–76. 10.1007/s10555-016-9629-x 27392603 PMC5035665

[99] Jahr S Hentze H Englisch S Hardt D Fackelmayer FO Hesch RD. DNA fragments in the blood plasma of cancer patients: quantitations and evidence for their origin from apoptotic and necrotic cells. Cancer Res. (2001) 61:1659–65. Available online at: https://cancerres.aacrjournals.org/content/61/4/1659.long 11245480

[100] Assou S Ait-Ahmed O El Messaoudi S Thierry AR Hamamah S. Non-invasive pre-implantation genetic diagnosis of X-linked disorders. Med Hypotheses. (2014) 83:506–8. 10.1016/j.mehy.2014.08.019 25182520

[101] Stroun M Lyautey J Lederrey C Olson-Sand A Anker P. About the possible origin and mechanism of circulating DNA apoptosis and active DNA release. Clin Chim Acta. (2001) 313:139–42. 10.1016/S0009-8981(01)00665-9 11694251

[102] Bettegowda C Sausen M Leary RJ Kinde I Wang Y Agrawal N. Detection of circulating tumor DNA in early- and late-stage human malignancies. Sci Transl Med. (2014) 6:224ra24. 10.1158/1538-7445.AM2014-5606 24553385 PMC4017867

[103] Phallen J Sausen M Adleff V Leal A Hruban C White J. Direct detection of early-stage cancers using circulating tumor DNA. Sci Transl Med. (2017) 9:eaan2415. 10.1126/scitranslmed.aan2415 28814544 PMC6714979

[104] Page K Powles T Slade MJ MT DEB Walker RA Coombes RC. The importance of careful blood processing in isolation of cell-free DNA. Ann N Y Acad Sci. (2006) 1075:313–7. 10.1196/annals.1368.042 17108226

[105] Hudecova I. Digital PCR analysis of circulating nucleic acids. Clin Biochem. (2015) 48:948–56. 10.1016/j.clinbiochem.2015.03.015 25828047

[106] Gale D Lawson ARJ Howarth K Madi M Durham B Smalley S. Development of a highly sensitive liquid biopsy platform to detect clinically-relevant cancer mutations at low allele fractions in cell-free DNA. PLoS ONE. (2018) 13:e0194630. 10.1371/journal.pone.0194630 29547634 PMC5856404

[107] Bahassi el M Stambrook PJ. Next-generation sequencing technologies: breaking the sound barrier of human genetics. Mutagenesis. (2014) 29:303–10. 10.1093/mutage/geu031 25150023 PMC7318892

[108] Slatko BE Gardner AF Ausubel FM. Overview of next-generation sequencing technologies. Curr Protoc Mol Biol. (2018) 122:e59. 10.1002/cpmb.59 29851291 PMC6020069

[109] Favero F Joshi T Marquard AM Birkbak NJ Krzystanek M Li Q. Sequenza: allele-specific copy number and mutation profiles from tumor sequencing data. Ann Oncol. (2015) 26:64–70. 10.1093/annonc/mdu479 25319062 PMC4269342

[110] Stasik S Schuster C Ortlepp C Platzbecker U Bornhäuser M Schetelig J. An optimized targeted next-generation sequencing approach for sensitive detection of single nucleotide variants. Biomol Detect Quantif. (2018) 15:6–12. 10.1016/j.bdq.2017.12.001 29349042 PMC5766748

[111] Diehl F Schmidt K Choti MA Romans K Goodman S Li M. Circulating mutant DNA to assess tumor dynamics. Nat Med. (2008) 14:985–90. 10.1038/nm.1789 18670422 PMC2820391

[112] Abbosh C Birkbak NJ Wilson GA Jamal-Hanjani M Constantin T Salari R. Phylogenetic ctDNA analysis depicts early-stage lung cancer evolution. Nature. (2017) 545:446–51. 10.1038/nature22364 28445469 PMC5812436

[113] Maron SB Chase LM Lomnicki S Kochanny S Moore KL Joshi SS. Circulating tumor DNA sequencing analysis of gastroesophageal adenocarcinoma. Clin Cancer Res. (2019) 25:7098–112. 10.1158/1078-0432.CCR-19-1704 31427281 PMC6891164

[114] Pandya D Camacho SC Padron MM Camacho-Vanegas O Billaud JN Beddoe AM. Rapid development and use of patient-specific ctDNA biomarkers to avoid a “rash decision” in an ovarian cancer patient. Cold Spring Harb Mol Case Stud. (2019) 5:a004648. 10.1101/mcs.a004648 31628202 PMC6913138

[115] Lin KK Harrell MI Oza AM Oaknin A Ray-Coquard I Tinker AV. BRCA reversion mutations in circulating tumor dna predict primary and acquired resistance to the PARP inhibitor rucaparib in high-grade ovarian carcinoma. Cancer Discov. (2019) 9:210–9. 10.1158/2159-8290.CD-18-0715 30425037

[116] Cheng X Zhang L Chen Y Qing C. Circulating cell-free DNA and circulating tumor cells, the “liquid biopsies” in ovarian cancer. J Ovarian Res. (2017) 10:75. 10.1186/s13048-017-0369-5 29132396 PMC5683341

[117] Chen Q Zhang ZH Wang S Lang JH. Circulating cell-Free DNA or circulating tumor DNA in the management of ovarian and endometrial cancer. Onco Targets Ther. (2019) 12:11517–30. 10.2147/OTT.S227156 31920340 PMC6938177

[118] Park YR Kim YM Lee SW Lee HY Lee GE Lee JE. Optimization to detect TP53 mutations in circulating cell-free tumor DNA from patients with serous epithelial ovarian cancer. Obstet Gynecol Sci. (2018) 61:328–36. 10.5468/ogs.2018.61.3.328 29780774 PMC5956115

[119] Swisher EM Wollan M Mahtani SM Willner JB Garcia R Goff BA. Tumor-specific p53 sequences in blood and peritoneal fluid of women with epithelial ovarian cancer. Am J Obstet Gynecol. (2005) 193:662–7. 10.1016/j.ajog.2005.01.054 16150257

[120] Parkinson CA Gale D Piskorz AM Biggs H Hodgkin C Addley H. Exploratory analysis of tp53 mutations in circulating tumour dna as biomarkers of treatment response for patients with relapsed high-grade serous ovarian carcinoma: a retrospective study. PLoS Med. (2016) 13:e1002198. 10.1371/journal.pmed.1002198 27997533 PMC5172526

[121] Vanderstichele A Busschaert P Smeets D Landolfo C Van Nieuwenhuysen E Leunen K. Chromosomal instability in cell-free DNA as a highly specific biomarker for detection of ovarian cancer in women with adnexal masses. Clin Cancer Res. (2017) 23:2223–31. 10.1158/1078-0432.CCR-16-1078 27852697

[122] Dobrzycka B Terlikowski SJ Mazurek A Kowalczuk O Niklinska W Chyczewski L. Circulating free DNA, p53 antibody and mutations of KRAS gene in endometrial cancer. Int J Cancer. (2010) 127:612–21. 10.1002/ijc.25077 19960433

[123] Pereira E Camacho-Vanegas O Anand S Sebra R Catalina Camacho S Garnar-Wortzel L. Personalized circulating tumor DNA biomarkers dynamically predict treatment response and survival in gynecologic cancers. PLoS ONE. (2015) 10:e0145754. 10.1371/journal.pone.0145754 26717006 PMC4696808

[124] Bolivar AM Luthra R Mehrotra M Chen W Barkoh BA Hu P. Targeted next-generation sequencing of endometrial cancer and matched circulating tumor DNA: identification of plasma-based, tumor-associated mutations in early stage patients. Mod Pathol. (2019) 32:405–414. 10.1038/s41379-018-0158-8 30315273 PMC6395490

[125] Tan DS Kaye SB. Chemotherapy for patients with BRCA1 and BRCA2-mutated ovarian cancer: same or different? Am Soc Clin Oncol Educ Book. (2015) 2015:114–21. 10.14694/EdBook_AM.2015.35.114 25993149

[126] Patch AM Christie EL Etemadmoghadam D Garsed DW George J Fereday S. Whole-genome characterization of chemoresistant ovarian cancer. Nature. (2015) 521:489–94. 10.1038/nature14410 26017449

[127] Christie EL Fereday S Doig K Pattnaik S Dawson SJ Bowtell DDL. Reversion of BRCA1/2 germline mutations detected in circulating tumor dna from patients with high-grade serous ovarian cancer. J Clin Oncol. (2017) 35:1274–80. 10.1200/JCO.2016.70.4627 28414925

[128] Cohen JD Li L Wang Y Thoburn C Afsari B Danilova L. Detection and localization of surgically resectable cancers with a multi-analyte blood test. Science. (2018) 359:926–30. 10.1126/science.aar3247 29348365 PMC6080308

[129] Wang Y Li L Douville C Cohen JD Yen TT Kinde I. Evaluation of liquid from the papanicolaou test and other liquid biopsies for the detection of endometrial and ovarian cancers. Sci Transl Med. (2018) 10:eaap8793. 10.1126/scitranslmed.aap8793 29563323 PMC6320220

[130] Lee RC Feinbaum RL Ambros VC. The elegans heterochronic gene lin-4 encodes small RNAs with antisense complementarity to lin-14. Cell. (1993) 75:843–54. 10.1016/0092-8674(93)90529-Y 8252621

[131] Ameres SL Zamore PD. Diversifying microRNA sequence and function. Nat Rev Mol Cell Biol. (2013) 14:475–88. 10.1038/nrm3611 23800994

[132] Friedman RC Farh KK Burge CB Bartel DP. Most mammalian mRNAs are conserved targets of microRNAs. Genome Res. (2009) 19:92–105. 10.1101/gr.082701.108 18955434 PMC2612969

[133] Arroyo JD Chevillet JR Kroh EM Ruf IK Pritchard CC Gibson DF. Argonaute2 complexes carry a population of circulating microRNAs independent of vesicles in human plasma. Proc Natl Acad Sci USA. (2011) 108:5003–8. 10.1073/pnas.1019055108 21383194 PMC3064324

[134] Tabet F Vickers KC Cuesta Torres LF Wiese CB Shoucri BM Lambert G. HDL-transferred microRNA-223 regulates ICAM-1 expression in endothelial cells. Nat Commun. (2014) 5:3292. 10.1038/ncomms4292 24576947 PMC4189962

[135] Van Roosbroeck K Calin GA. Cancer hallmarks and microRNAs: the therapeutic connection. Adv Cancer Res. (2017) 135:119–49. 10.1016/bs.acr.2017.06.002 28882220

[136] Mitchell PS Parkin RK Kroh EM Fritz BR Wyman SK Pogosova-Agadjanyan EL. Circulating microRNAs as stable blood-based markers for cancer detection. Proc Natl Acad Sci USA. (2008) 105:10513–8. 10.1073/pnas.0804549105 18663219 PMC2492472

[137] Umu SU Langseth H Bucher-Johannessen C Fromm B Keller A Meese E. A comprehensive profile of circulating RNAs in human serum. RNA Biol. (2018) 15:242–50. 10.1080/15476286.2017.1403003 29219730 PMC5798962

[138] Nakamura K Sawada K Yoshimura A Kinose Y Nakatsuka E Kimura T. Clinical relevance of circulating cell-free microRNAs in ovarian cancer. Mol Cancer. (2016) 15:48. 10.1186/s12943-016-0536-0 27343009 PMC4921011

[139] Souza MF Kuasne H Barros-Filho MC Ciliao HL Marchi FA Fuganti PE. Circulating mRNAs and miRNAs as candidate markers for the diagnosis and prognosis of prostate cancer. PLoS ONE. (2017) 12:e0184094. 10.1371/journal.pone.0184094 28910345 PMC5598937

[140] Wang H Peng R Wang J Qin Z Xue L. Circulating microRNAs as potential cancer biomarkers: the advantage and disadvantage. Clin Epigenetics. (2018) 10:59. 10.1186/s13148-018-0492-1 29713393 PMC5913875

[141] Zheng H Zhang L Zhao Y Yang D Song F Wen Y. Plasma miRNAs as diagnostic and prognostic biomarkers for ovarian cancer. PLoS ONE. (2013) 8:e77853. 10.1371/journal.pone.0077853 24223734 PMC3815222

[142] Wang L Chen YJ Xu K Xu H Shen XZ Tu RQ. Circulating microRNAs as a fingerprint for endometrial endometrioid adenocarcinoma. PLoS ONE. (2014) 9:e110767. 10.1371/journal.pone.0110767 25329674 PMC4203829

[143] Giannopoulou L Zavridou M Kasimir-Bauer S Lianidou ES. Liquid biopsy in ovarian cancer: the potential of circulating miRNAs and exosomes. Transl Res. (2019) 205:77–91. 10.1016/j.trsl.2018.10.003 30391474

[144] Shih CL Luo JD Chang JW Chen TL Chien YT Yu CJ. Circulating messenger rna profiling with microarray and next-generation sequencing: cross-platform comparison. Cancer Genomics Proteomics. (2015) 12:223–30. Available online at: http://cgp.iiarjournals.org/content/12/5/223.long 26417025

[145] EL Andaloussi S Mäger I Breakefield XO Wood MJ. Extracellular vesicles: biology and emerging therapeutic opportunities. Nat Rev Drug Discov. (2013) 12:347–57. 10.1038/nrd3978 23584393

[146] Valadi H Ekstrom K Bossios A Sjostrand M Lee JJ Lotvall JO. Exosome-mediated transfer of mRNAs and microRNAs is a novel mechanism of genetic exchange between cells. Nat Cell Biol. (2007) 9:654–9. 10.1038/ncb1596 17486113

[147] Greening DW Gopal SK Xu R Simpson RJ Chen W. Exosomes and their roles in immune regulation and cancer. Semin Cell Dev Biol. (2015) 40:72–81. 10.1016/j.semcdb.2015.02.009 25724562

[148] Torralba D Baixauli F Villarroya-Beltri C Fernandez-Delgado I Latorre-Pellicer A Acin-Perez R. Priming of dendritic cells by DNA-containing extracellular vesicles from activated T cells through antigen-driven contacts. Nat Commun. (2018) 9:2658. 10.1038/s41467-018-05077-9 29985392 PMC6037695

[149] Lee Y El Andaloussi S Wood MJ. Exosomes and microvesicles: extracellular vesicles for genetic information transfer and gene therapy. Hum Mol Genet. (2012) 21(R1):R125–34. 10.1093/hmg/dds317 22872698

[150] Skog J T. Wurdinger van Rijn S Meijer DH Gainche L Sena-Esteves. Glioblastoma microvesicles transport RNA and proteins that promote tumour growth and provide diagnostic biomarkers. Nat Cell Biol. (2008) 10:1470–6. 10.1038/ncb1800 19011622 PMC3423894

[151] Endzelins E Berger A Melne V Bajo-Santos C Sobolevska K Abols A. Detection of circulating miRNAs: comparative analysis of extracellular vesicle-incorporated miRNAs and cell-free miRNAs in whole plasma of prostate cancer patients. BMC Cancer. (2017) 17:730. 10.1186/s12885-017-3737-z 29121858 PMC5679326

[152] Qi J Zhou Y Jiao Z Wang X Zhao Y Li Y. Exosomes derived from human bone marrow mesenchymal stem cells promote tumor growth through hedgehog signaling pathway. Cell Physiol Biochem. (2017) 42:2242–54. 10.1159/000479998 28817816

[153] Zhu G Pei L Lin F Yin H Li X He W. Exosomes from human-bone-marrow-derived mesenchymal stem cells protect against renal ischemia/reperfusion injury via transferring miR-199a-3p. J Cell Physiol. (2019) 234:23736–49. 10.1002/jcp.28941 31180587

[154] Herrero C de la Fuente A Casas-Arozamena C Sebastian V Prieto M Arruebo M. Extracellular vesicles-based biomarkers represent a promising liquid biopsy in endometrial cancer. Cancers. (2019) 11:2000. 10.3390/cancers11122000 31842290 PMC6966595

[155] Urabe F Kosaka N Ito K Kimura T Egawa S Ochiya T. Extracellular vesicles as biomarkers and therapeutic targets for cancer. Am J Physiol Cell Physiol. (2020) 318:C29–c39. 10.1152/ajpcell.00280.2019 31693397

[156] Whiteside TL. The potential of tumor-derived exosomes for noninvasive cancer monitoring: an update. Expert Rev Mol Diagn. (2018) 18:1029–40. 10.1080/14737159.2018.1544494 30406709 PMC6506389

[157] Li W Li C Zhou T Liu X Liu X Li X. Role of exosomal proteins in cancer diagnosis. Mol Cancer. (2017) 16:145. 10.1186/s12943-017-0706-8 28851367 PMC5576100

[158] Su YY Sun L Guo ZR Li JC Bai TT Cai XX. Upregulated expression of serum exosomal miR-375 and miR-1307 enhance the diagnostic power of CA125 for ovarian cancer. J Ovarian Res. (2019) 12:6. 10.1186/s13048-018-0477-x 30670062 PMC6341583

[159] Tang MKS Yue PYK Ip PP Huang RL Lai HC Cheung ANY. Soluble E-cadherin promotes tumor angiogenesis and localizes to exosome surface. Nat Commun. (2018) 9:2270. 10.1038/s41467-018-04695-7 29891938 PMC5995921

[160] Nakamura K Sawada K Kinose Y Yoshimura A Toda A Nakatsuka E. Exosomes promote ovarian cancer cell invasion through transfer of cd44 to peritoneal mesothelial cells. Mol Cancer Res. (2017) 15:78–92. 10.1158/1541-7786.MCR-16-0191 27758876

[161] Zhou J Li X Wu X Zhang T Zhu Q Wang X. Exosomes released from tumor-associated macrophages transfer mirnas that induce a Treg/Th17 cell imbalance in epithelial ovarian cancer. Cancer Immunol Res. (2018) 6:1578–92. 10.1158/2326-6066.CIR-17-0479 30396909

[162] Mavaddat N Peock S Frost D Ellis S Platte R Fineberg E. Cancer risks for BRCA1 and BRCA2 mutation carriers: results from prospective analysis of EMBRACE. J Natl Cancer Inst. (2013) 105:812–22. 10.1093/jnci/djt095 23628597

